# A case of intrascrotal extratesticular schwannoma

**DOI:** 10.1016/j.radcr.2023.07.029

**Published:** 2023-07-22

**Authors:** Ryo Takaji, Satoki Abe, Toshitaka Shin, Tsutomu Daa, Ryuichi Shimada, Yoshiki Asayama

**Affiliations:** a Department of Radiology, Oita University Faculty of Medicine, Yufu, Oita, Japan; b Department of Urology, Oita University Faculty of Medicine, Yufu, Oita, Japan; c Department of Diagnostic Pathology, Oita University Faculty of Medicine, Yufu, Oita, Japan

**Keywords:** Scrotal tumor, Schwannoma, Tunica albuginea, Magnetic resonance imaging

## Abstract

Schwannomas are benign tumors arising from Schwann cells, which compose the myelin sheath covering peripheral nerves. Although schwannomas can develop in various locations throughout the human body, the scrotum is a rare site for development of a schwannoma. Furthermore, to the best of our knowledge, no study to date has focused on the detailed imaging findings of intrascrotal schwannoma.

## Introduction

Schwannomas are benign tumors that arise from Schwann cells of peripheral nerve sheaths and can occur at any age without a sex predilection, either sporadically or in association with neurofibromatosis type 2 and schwannomatosis [1,2]. Although schwannomas commonly occur in the head and neck region and the extremities, they can also develop in various other locations throughout the body [3], including the posterior mediastinum, retroperitoneum, spinal cord, bones, gastrointestinal tract, pancreas, liver, thyroid, adrenal glands, and other sites. Schwannomas originating within the scrotum are rare, and within the scope of our search, we found only 14 reported cases [4], [5], [6], [7], [8], [9], [10], [11], [12], [13], [14], [15], [16], [17], [18]. We herein present a case of an intrascrotal extratesticular schwannoma with a focus on magnetic resonance imaging (MRI) features.

## Case report

A 41-year-old man presented with discomfort in the left scrotum, and an ultrasound examination revealed a mass within the left scrotum. He was then referred to our urology department because of suspicion of a left testicular tumor. The patient had a history of schwannomas in the left forearm 14 years previously and in the right thigh 6 years previously. Blood biochemical tests revealed no significant findings. The serum level of lactate dehydrogenase was 167 U/L, alpha-fetoprotein was 4.27 ng/mL, squamous cell carcinoma antigen was 0.8 ng/mL, interleukin 2 receptor was 363 U/mL, and human chorionic gonadotropin was <0.5 mIU/mL, all of which were within the normal range.

**Fig. 1:**
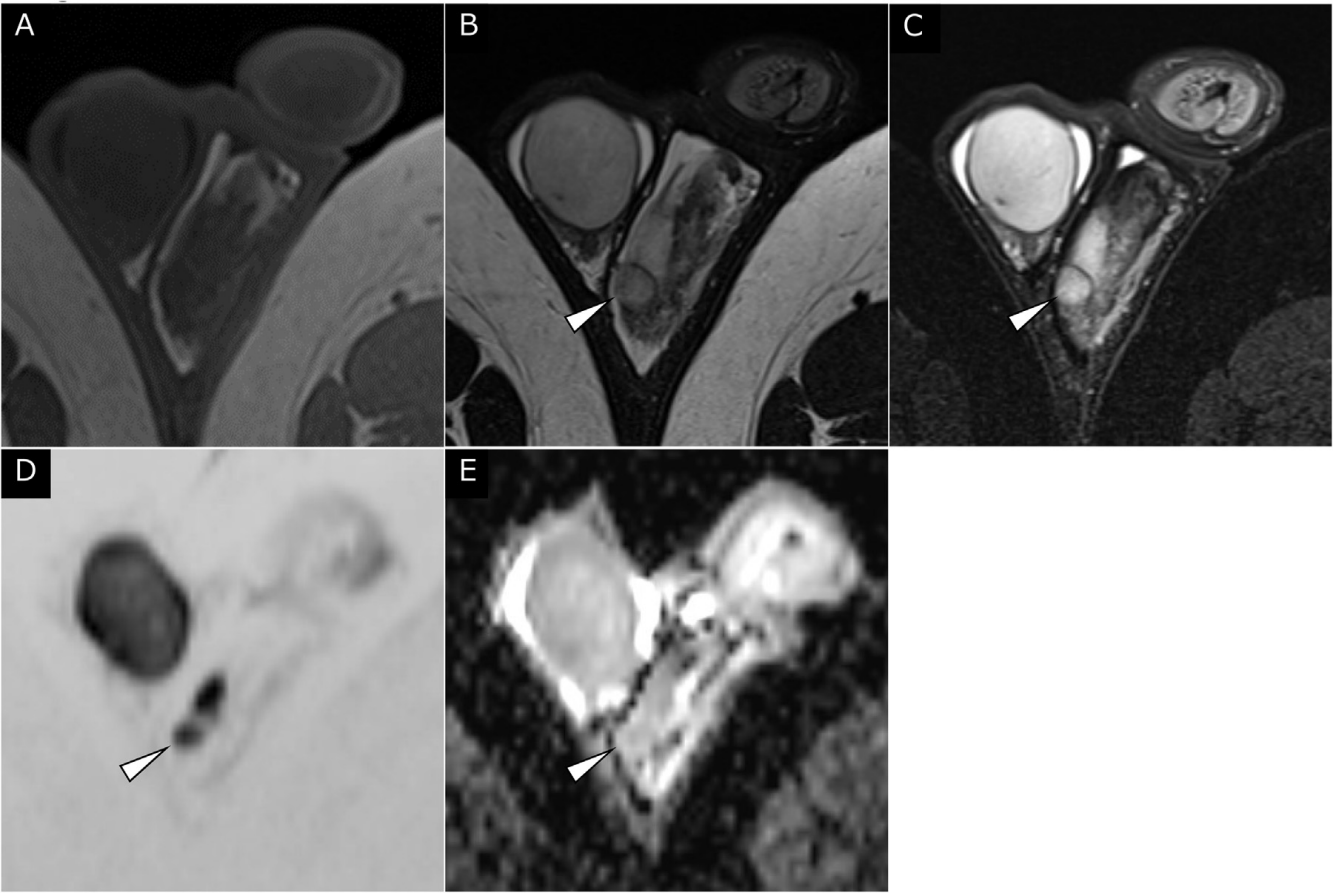
Axial MRI of the left scrotal mass. (A) T1WI failed to demonstrate the tumor. A well-defined slightly inhomogeneous 10-mm mass of high signal intensity (arrowheads) was present at the periphery of the left testis on (B) T2WI and (C) fat-suppressed T2WI. (D, E) On DWI, the mass (arrowheads) showed diffusion restriction comparable to that of the normal testis (apparent diffusion coefficient of the mass: 1.1 × 10^−3^ mm^2^/s).

**Fig. 2:**
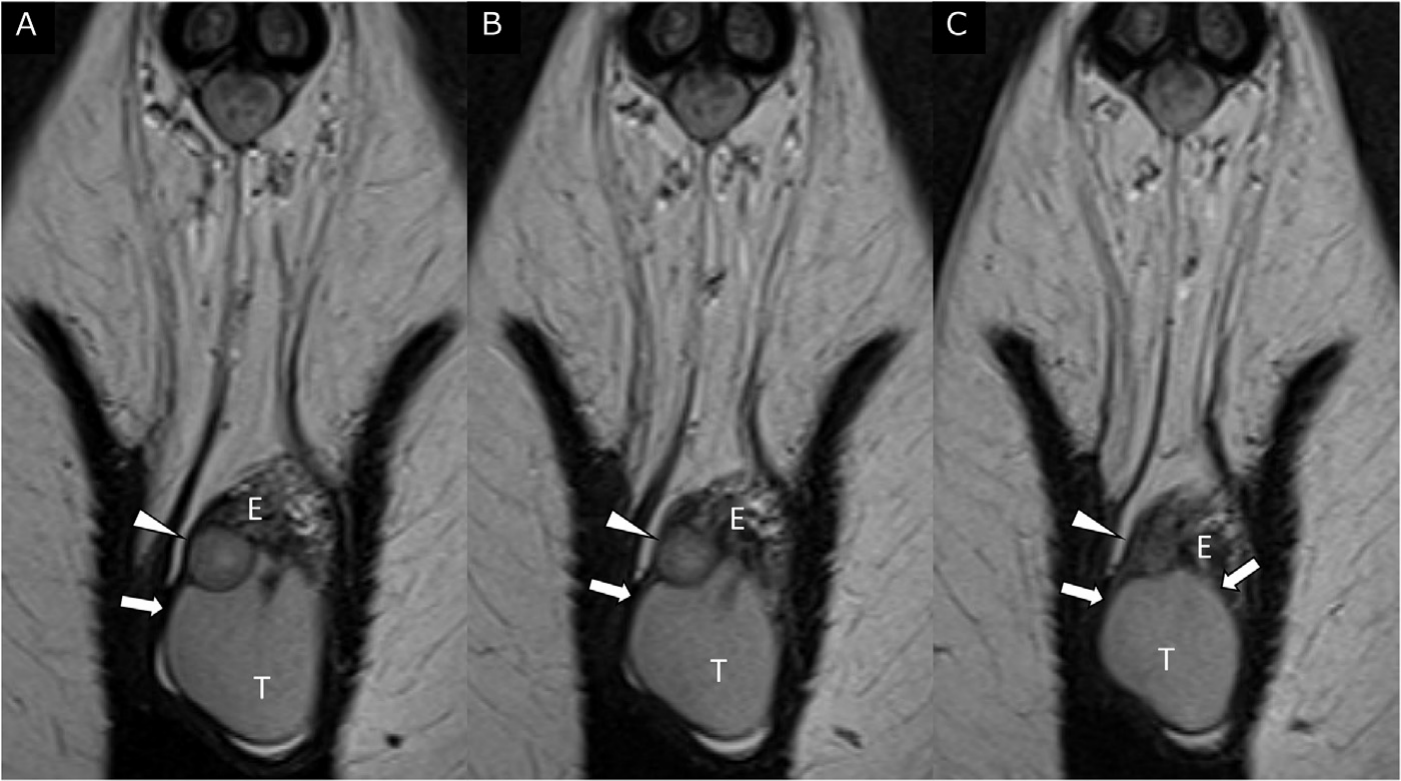
Coronal T2WI of the left scrotal mass. (A–C) A small mass with a rim of low signal intensity (arrowheads) was depicted between the left testis (T) and epididymis (E). The low-signal-intensity rim had continuity with the tunica albuginea and vaginalis (arrows). The mass showed similar or slightly lower signal intensity compared with the normal testis.

**Fig. 3:**
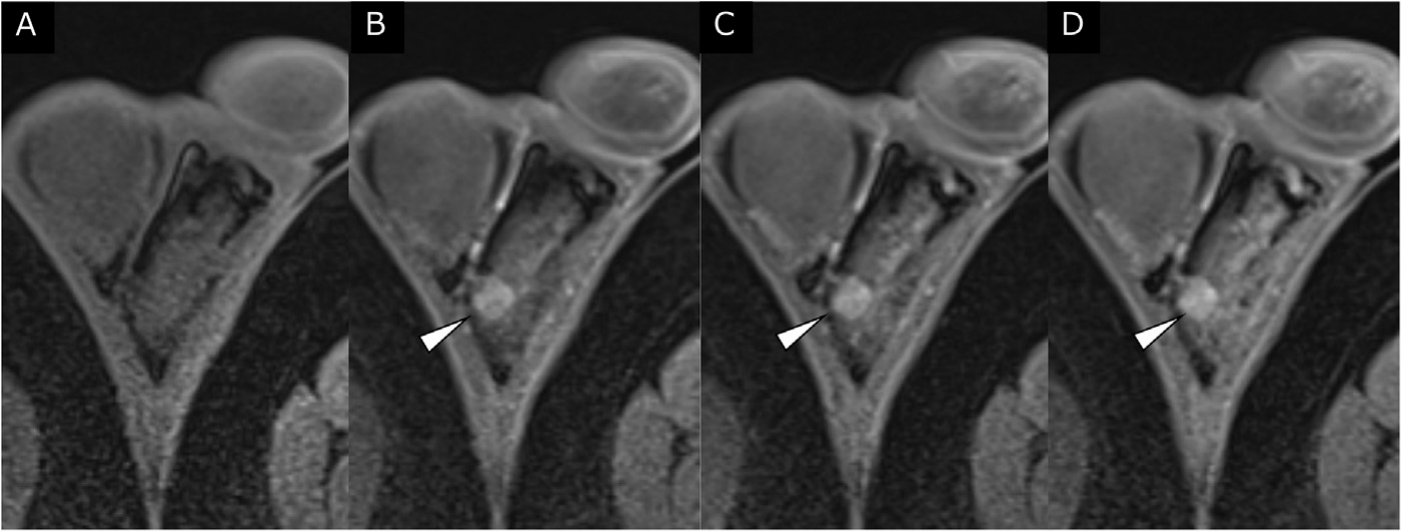
Dynamic contrast-enhanced MRI, axial images. **(**A) Precontrast fat-suppressed T1WI failed to demonstrate the tumor. The mass showed inhomogeneous contrast enhancement on postcontrast scans at (B) 40 seconds, (C) 70 seconds, and (D) 150 seconds after intravenous gadolinium injection.

MRI revealed a left scrotal mass measuring 1 cm. The mass showed isointensity compared with the normal testis on T1-weighted imaging (T1WI) (Fig. 1). A well-defined, slightly inhomogeneous mass with high signal intensity was demonstrated between the left testis and epididymis on T2WI (Figs. 1 and 2). On T2WI, the mass had a rim with low signal intensity and continuity with the tunica albuginea and vaginalis (Figs. 1 and 2). On diffusion-weighted imaging (DWI) (Fig. 1), the mass showed diffusion restriction comparable to that of the normal testis (apparent diffusion coefficient of the mass: 1.1*10^−3^mm^2^/sec). On dynamic contrast-enhanced MRI obtained 40, 70, and 150 seconds after intravenous gadolinium injection (Fig. 3), the mass showed inhomogeneous contrast enhancement on each postcontrast scan.

A benign tumor derived from the tunica albuginea or tunica vaginalis, such as an adenomatoid tumor, was considered as a preoperative imaging diagnosis. However, it was not possible to rule out a testicular tumor arising from the peripheral region of the testis, and a high orchiectomy was therefore performed.

**Fig. 4:**
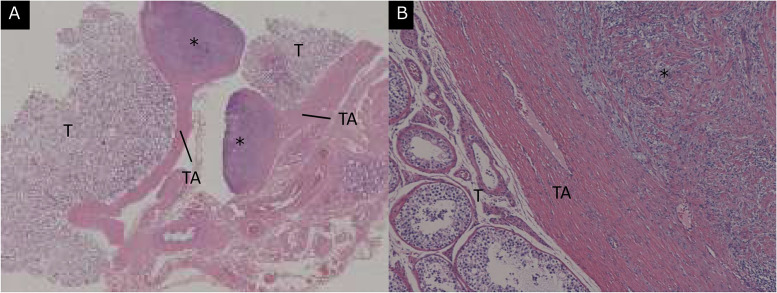
Histological section (hematoxylin and eosin stain) of the scrotal tumor. (A) Pathologically, the tumor (*) showed continuity with the tunica albuginea (TA) and was adjacent to the left testis (T). (B) No abnormal findings were observed in the left testis and epididymis.

**Fig. 5:**
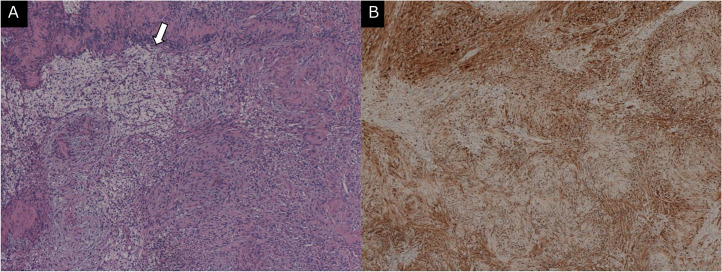
Histological sections (hematoxylin and eosin stain and immunostaining for S-100) of the scrotal tumor. (A) On hematoxylin and eosin staining, the tumor consisted of spindle cell proliferation without an increase in mitotic figures. No intratumoral hemorrhage or necrosis was observed. A component consisting of spindle cells with a loose myxoid matrix (arrow) was partially confirmed. (B) The tumor cells showed intense immunoreactivity for S-100 staining. A paratesticular schwannoma was pathologically confirmed.

Macroscopically, a well-circumscribed tumor was found adjacent to the left testis. Microscopically, the tumor had continuity with the tunica albuginea, and no abnormal findings were observed in the left testis or epididymis (Fig. 4). The tumor consisted of spindle cell proliferation without an increase in mitotic figures and a loose myxoid matrix (Fig. 5). No intratumoral hemorrhage or necrosis was observed. The tumor cells showed intense immunoreactivity for S-100 staining (Fig. 5). A paratesticular schwannoma was pathologically confirmed.

After the testicular surgery, the patient developed left popliteal pain, and MRI revealed a mass in the left popliteal fossa with characteristics suggestive of a schwannoma.

## Discussion

Schwannomas originating within the scrotum are rare, and within the scope of our search, we found only 14 reported cases [4–18]. The clinical and imaging findings of these previously reported cases and the current case are summarized in Table 1.

**Table 1:** Summary of previously reported intrascrotal schwannomas and our case.

Case	Age (yr)	Clinical symptoms	Tumor marker	Size (cm)	Side	Location	Imaging findings	Diagnosis
4)	52	Painless scrotal mass	Negative	7	Left	Extratesticular	Hypoechoic (US)	Schwannoma
5)	79	Painless scrotal mass	Negative	1	Right	Itratesticular, tunica albuginea involvement	Hypoechoic (US)	Schwannoma
6)	28	Painless scrotal mass	NA	7	Middle	Extratesticular	Multinodular cystic mass (US)	Ancient schwannoma
7)	53	Painless scrotal mass	NA	6	Right	Extratesticular	NA	Schwannoma
8)	58	Painless scrotal mass	NA	20	Left	Extratesticular	Heterogeneous (US)	Schwannoma
9)	67	Painless scrotal mass	Negative	3.5	Middle	Extratesticular	Hypo-hyperechoic (US)	Multiple schwannoma
10)	70	Painless scrotal mass	Negative	7.5	Left	Extratesticular	Heterogeneous, cystic (US)	Schwannoma
11, 12)	48	Painless scrotal mass	NA	7	Left	Extratesticular	Heterogeneous, solid (US)	Malignant schwannoma
13)	38	Painless scrotal mass	Negative	2.3	Left	Extratesticular	Heterogeneous (US), low T1/heterogeneous T2 SI, heterogeneous enhanced (MR)	Schwannoma
14)	45	Painless scrotal mass	Negative	9.5	Left	Extratesticular	Heterogeneous, (US)	Schwannoma
15)	66	Painless scrotal mass	NA	3	Middle	Extratesticular	Peripheral enhancement (MR)	Schwannoma, Schwannomatosis
16)	71	Painless scrotal mass	NA	3	Right	Extratesticular	Hyperechoic (US), calcification (CT), iso T1/ high T2 SI (MR)	Schwannoma
17)	65	Painless scrotal mass	NA	2.8	Right	Extratesticular	NA	Schwannoma
18)	56	Painless scrotal mass	Negative	1.8	Left	Extratesticular	Heterogeneous, cystic change, calcification (US)	Schwannoma
Our case	41	Painless scrotal mass	Negative	1	Left	Extratesticular, tunica albuginea	low T1/heterogeneous T2 SI, diffusion restriction, heterogeneous enhanced (MR)	Schwannoma, possibility of schwannomatosis

CT, computed tomography; MR, magnetic resonance imaging; NA, Not applicable; SI, signal intensity; US, ultrasonography.

The previously reported cases [4–18] of scrotal schwannoma had an average patient age of 56.9 years (range, 28-79 years) and an average tumor size of 5.8 cm (range, 1-20 cm). In all cases, the main complaint was painless scrotal swelling. There were no specific elevations of serum testicular tumor markers, and the patients exhibited nonspecific clinical symptoms. With respect to tumor localization, 7 cases originated from the left side, 3 from the middle portion, and 4 from the right side [4–18]. Thirteen cases were intrascrotal extratesticular lesions [4,^6^–18], and 1 lesion was intratesticular in origin and involved tunica albuginea [5]. In our case, a man in his 40s presented with scrotal discomfort, negative serum tumor markers, and nonspecific clinical findings. The lesion was located within the left scrotum and was 1 cm in size, which is relatively small compared with previously reported cases. It was an extratesticular lesion located at the periphery of the testis and continuous with the tunica albuginea.

No reports to date have described the detailed imaging findings of intrascrotal schwannomas. In the present case, MRI depicted a well-defined paratesticular tumor with suspected involvement of the tunica albuginea and tunica vaginalis, with a heterogeneous internal signal on T2WI and diffusion restriction similar to that of the normal testis on DWI. On dynamic contrast-enhanced MRI, the tumor showed heterogeneous enhancement on each postcontrast image. Common MRI findings of schwannoma include a well-defined tumor, the “target sign” on T2WI (reflecting a high signal at the periphery corresponding to Antoni B-type cells and a low signal in the central region corresponding to Antoni A-type cells), the “split fat sign,” and heterogeneous enhancement on contrast-enhanced MRI [19]. DWI typically shows restricted diffusion. In retrospect, the MRI findings in the present case were consistent with a schwannoma, although an intrascrotal schwannoma is very rare.

Localization is crucial for determining the benign or malignant nature of intrascrotal tumors. Whereas many testicular tumors are malignant, most intrascrotal extratesticular tumors are considered benign (such as lipomas, leiomyomas, and adenomatoid tumors) [20,21]. In our case, based on preoperative T2WI, we suspected an extratesticular lesion; however, because of the small size of the lesion, we could not exclude the possibility of a testicular tumor originating from the peripheral testicular tissue. Additionally, the presence of a tumor in the tunica albuginea might make accurate localization difficult. Dynamic contrast-enhanced MRI is also useful for determining the benign or malignant nature of intrascrotal tumors, and evaluation based on time–intensity curves created by tracking the enhancement for up to 8 minutes after contrast administration is recommended [21]. In the present case, the imaging time of contrast-enhanced dynamic MRI was 150 seconds after contrast agent injection, which might have been insufficient for a definitive assessment of benign or malignant characteristics.

Schwannomas can sometimes occur as multiple lesions, and in patients with multiple schwannomas, the possibility of neurofibromatosis type 2 or schwannomatosis should be considered. Cases of schwannomatosis associated with intrascrotal schwannoma have also been reported [15]. In the present case, the patient had a surgical history of schwannoma in his limbs 14 years and 6 years prior to the testicular surgery. Additionally, after the testicular surgery, the patient developed left popliteal pain, and MRI revealed a tumor in the left popliteal fossa that was suspected to be a schwannoma. Although a search for intracranial lesions was not performed, the possibility of schwannomatosis should be considered in such cases.

Schwannomas are usually benign and can be treated with tumor resection. While reports of malignant schwannoma with rhabdomyosarcoma-like changes exist [11,12], favorable postoperative outcomes have been reported [12]. In the present case, high orchiectomy was performed. However, if the diagnosis of schwannoma had been made preoperatively, less invasive surgery might have been considered as a treatment option.

## Conclusions

We experienced a case of intrascrotal extratesticular schwannoma. The lesion appeared within the scrotum and extended from the tunica albuginea, and it exhibited MRI findings consistent with a typical schwannoma. The occurrence of intrascrotal schwannoma is rare; however, when an intrascrotal tumor exhibits encapsulated heterogeneous T2-weighted signal intensity and contrast enhancement with diffusion restriction, the possibility of schwannoma should be considered.

## Author contributions

Ryo Takaji: Case file retrieval and case summary preparation. Ryo Takaji and Yoshiki Asayama: preparation of manuscript and editing. All authors provided final approval of the submitted version.

## Patient consent

Informed consent was obtained from the patient for the publication of this report and any accompanying images.
